# Establishment of bone marrow-derived M-CSF receptor-dependent self-renewing macrophages

**DOI:** 10.1038/s41420-020-00300-3

**Published:** 2020-07-23

**Authors:** Hesham Nasser, Partho Adhikary, Amira Abdel-Daim, Osamu Noyori, Jutatip Panaampon, Ryusho Kariya, Seiji Okada, Wenjuan Ma, Masaya Baba, Hitoshi Takizawa, Mariko Yamane, Hitoshi Niwa, Shinya Suzu

**Affiliations:** 1 Joint Research Center for Human Retrovirus Infection, Kumamoto University, Kumamoto, 860-0811 Japan; 2 International Research Center for Medical Sciences, Kumamoto University, Kumamoto, 860-0811 Japan; 3 Department of Clinical Pathology, Faculty of Medicine, Suez Canal University, Ismailia, 41511 Egypt; 4 Center for Metabolic Regulation of Healthy Aging, Kumamoto University, Kumamoto, 860-8556 Japan; 5 Institute of Molecular Embryology and Genetics, Kumamoto University, Kumamoto, 860-0811 Japan; 6 Present Address: Faculty of Pharmaceutical Sciences, The University of British Columbia, Vancouver, V6T 1Z3 Canada; 7 Present Address: Laboratory for Bioinformatics Research, RIKEN Center for Biosystems Dynamics Research, Hyogo, 650-0047 Japan

**Keywords:** Cell growth, Innate immune cells

## Abstract

Recent studies have revealed that tissue macrophages are derived from yolk sac precursors or fetal liver monocytes, in addition to bone marrow monocytes. The relative contribution of these cells to the tissue macrophage pool is not fully understood, but embryo-derived cells are supposed to be more important because of their capacity to self-renew. Here, we show the presence of adult bone marrow-derived macrophages that retain self-renewing capacity. The self-renewing macrophages were readily obtained by long-term culture of mouse bone marrow cells with macrophage colony-stimulating factor (M-CSF), a key cytokine for macrophage development. They were non-tumorigenic and proliferated in the presence of M-CSF in unlimited numbers. Despite several differences from non-proliferating macrophages, they retained many features of cells of the monocytic lineage, including the differentiation into dendritic cells or osteoclasts. Among the transcription factors involved in the self-renewal of embryonic stem cells, Krüppel-like factor 2 (KLF2) was strongly upregulated upon M-CSF stimulation in the self-renewing macrophages, which was accompanied by the downregulation of MafB, a transcription factor that suppresses KLF2 expression. Indeed, knockdown of KLF2 led to cell cycle arrest and diminished cell proliferation in the self-renewing macrophages. Our new cell model would be useful to unravel differences in phenotype, function, and molecular mechanism of proliferation among self-renewing macrophages with different origins.

## Introduction

It was believed that tissue-resident macrophages in an adult are maintained by a constant replenishment by bone marrow-derived circulating monocytes in the steady state, and the monocyte-derived macrophages are non-proliferating cells^1^. However, recent studies have shown that macrophages in several tissues were derived from the precursors in yolk sac or fetal liver independently of bone marrow, and self-maintained throughout life^2,3^. Although the relative contribution of these precursors in the yolk sac, fetal liver, or bone marrow to maintain macrophage pool in each tissue is not fully understood^4^, embryo-derived counterparts are supposed to be more important because of their self-renewing ability.

Nevertheless, several in vivo studies using mice have demonstrated that bone marrow-derived monocytes can also differentiate into self-renewing tissue-resident macrophages that resemble their embryonic counterparts, under certain circumstances^5–8^. For instance, multiple fate-mapping approaches showed that arterial macrophages arise embryonically from CX3CR1^+^ precursors and postnatally from bone marrow-derived monocytes that colonize the tissue immediately after birth, and the proliferation of the two populations sustains arterial macrophages in the steady state and after severe depletion of sepsis^8^. Thus, it appears that the pool of tissue macrophages can be maintained by the recruitment of monocytes, and the proliferation of both embryo-derived macrophages and bone marrow-derived macrophages^9–11^. However, it remains unclear how the two self-renewing populations are similar and different in phenotype and function, and whether they possess an equal self-renewing ability or share a common mechanism of self-renewal. To this end, the isolation, expansion and ex vivo analysis of these populations are required.

Fejer et al. recently reported the isolation and expansion of mouse fetal liver-derived self-renewing macrophages, which proliferate in vitro for an extended period in almost unlimited numbers in the presence of a cytokine, granulocyte/macrophage colony-stimulating factor (GM-CSF)^12^. The fetal liver-derived macrophages (MPI cells) also proliferate in the presence of another cytokine, macrophage CSF (M-CSF, also known as CSF-1), albeit in a slower rate than the presence of GM-CSF^12^. The isolation and expansion of mouse yolk sac-derived M-CSF-dependent macrophages have also been reported^13^. However, it is not well understood what molecular mechanisms enable the embryo-derived self-renewing macrophage model to proliferate for a long period in the presence of cytokines, in particular, M-CSF, a key regulator for macrophage development in most tissues^14,15^. In addition, the isolation and expansion of an adult counterpart, that is, bone marrow-derived M-CSF-dependent self-renewing macrophage cell model has not been reported yet.

Here, we report a simple culture method to enrich and expand M-CSF-dependent self-renewing macrophages from mouse bone marrow, and their phenotypical and functional characteristics. Furthermore, we provide evidence suggesting that their self-renewal is governed by molecular machinery that involves transcription factors, Krüppel-like factor 2 (KLF2) and c-Myc, as proposed in an engineered self-renewing macrophage model (MafB/c-Maf double knockout (Maf-DKO) macrophages) established from mice with combined deficiency for other transcription factors, MafB and c-Maf^16,17^.

## Results

### Expansion of bone marrow self-renewing macrophages in a long-term culture

**Fig. 1:**
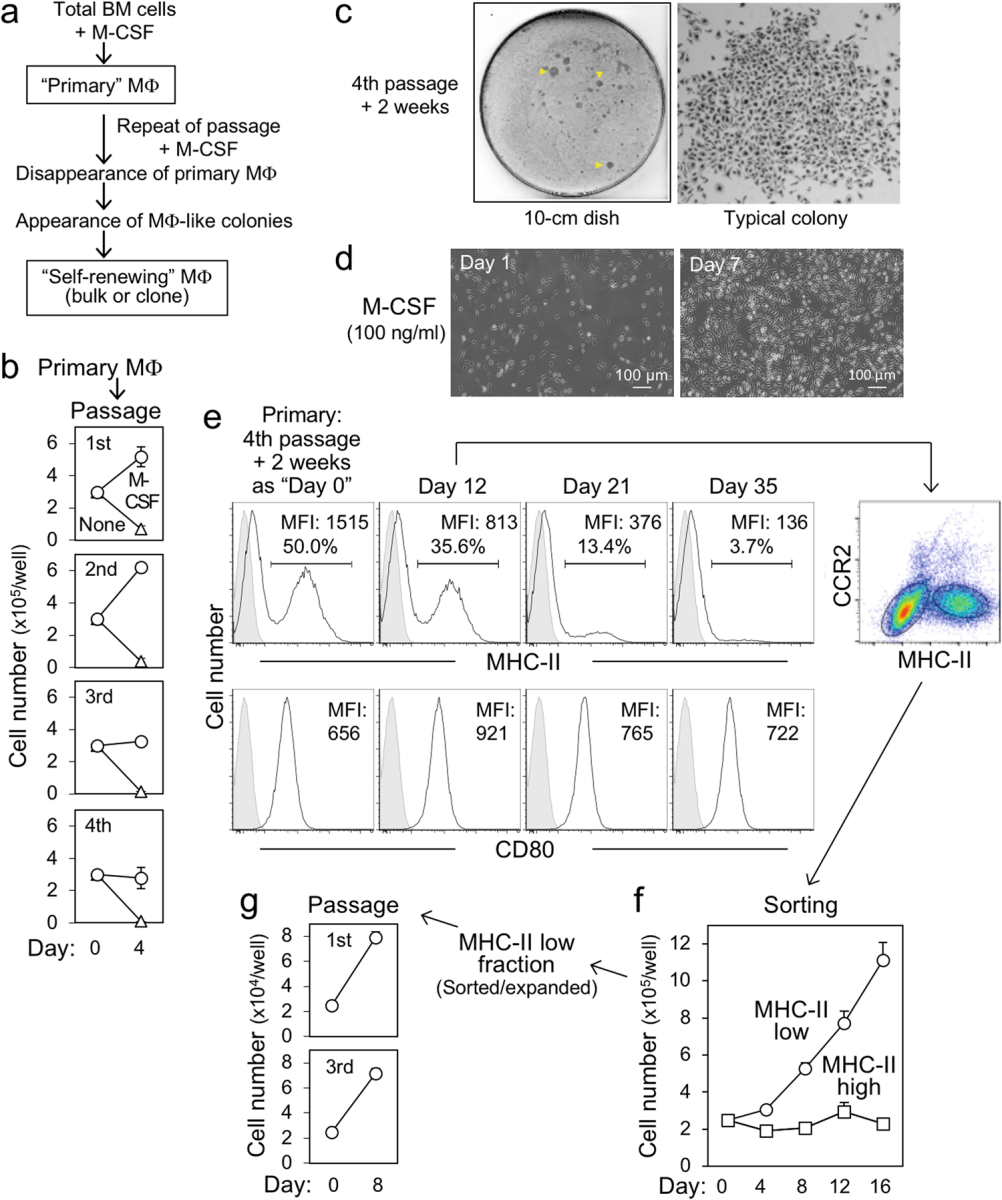
Expansion of the self-renewing macrophages in the long-term culture. **a** The outline of the long-term culture with a repeated passage. BM bone marrow, MΦ macrophages. **b** Primary bone marrow-derived macrophages were detached, seeded at 3 × 10^5^ cells/well, and cultured for 4 days in the absence (none) or presence of 100 ng/ml rhM-CSF. The cells were serially passaged and the number of viable cells was counted (mean ± SD, *n* = 3). **c** After the fourth passage, cells were cultured for 2 weeks in the presence of rhM-CSF and stained by Wright-Giemsa staining. Colonies appeared are indicated by yellow arrowheads (left panel). **d** Colonies appeared as in **c** were collected, reseeded, and cultured for 7 days in the presence of rhM-CSF. **e** After the fourth passage, cells were cultured for 2 weeks in the presence of rhM-CSF (defined as “day 0”) and analyzed for the expression of MHC-II by flow cytometry. CD80 was added as a reference. The analysis was repeated as indicated. At day 12, cells were also analyzed for the expression of CCR2. The mean fluorescence intensity (MFI) and the percentage of positive cells (for MHC-II) are shown. **f** The MHC-II^low^ and MHC-II^high^ fraction observed as in **e** (day 12) were sorted and cultured for up to day 16 in the presence of rhM-CSF, and the number of viable cells was counted (mean ± SD, *n* = 3). **g** The MHC-II^low^ cells expanded in **f** were reseeded and cultured for 8 days in the presence of rhM-CSF. The cells were serially passaged and the number of viable cells was counted (mean ± SD, *n* = 3).

We hypothesized that if macrophages with self-renewing ability are present in bone marrow, they can be expanded during a long-term culture. We therefore cultured bone marrow cells of C57BL/6 mice in the presence of M-CSF, and repeated detachment and reseeding of macrophages (Fig. 1a). Adherent cells after the initial seeding of bone marrow cells were defined as primary macrophages. The proliferation of primary macrophages became slower along with the passage, and most macrophages ceased to proliferate and begun to die at the fourth passage (Fig. 1b). Instead, several growing colonies appeared (Fig. 1c, left), which were composed of macrophage-like adherent cells (right). Cells in the colonies proliferated when isolated and reseeded (Fig. 1d). Similar proliferating macrophage-like cells were obtained when the bone marrow of BALB/c mice was used (Supplementary Fig. 1), indicating that their presence is independent of mouse strains. Of interest, cells in the fourth passage colonies contained two distinct populations based on the expression level of MHC class II (Fig. 1e, upper). MHC-II^low^ population predominated in the culture over time (Fig. 1e, upper), but no such change was observed for CD80 (lower). Self-renewing macrophages derived from fetal liver express MHC-II at a low level^12^, and monocyte-derived macrophages in the heart express a chemokine receptor CCR2 at a high level^9,18^. In line with these findings, the MHC-II^low^ population expressed CCR2 at a lower level than the MHC-II^high^ population (Fig. 1e, right). Moreover, when purified, the MHC-II^low^ population, but not the MHC-II^high^ population, proliferated (Fig. 1f) and could be serially passaged (Fig. 1g). Thus, it is highly likely that during the long-term culture with repeated passages, monocyte-derived MHC-II^high^ macrophages disappeared gradually because of a limited ability to divide, whereas MHC-II^low^ macrophage population with self-renewing capacity in the bone marrow survived and expanded.

### Non-tumorigenicity and M-CSF receptor-dependent proliferation of the self-renewing macrophages

**Fig. 2:**
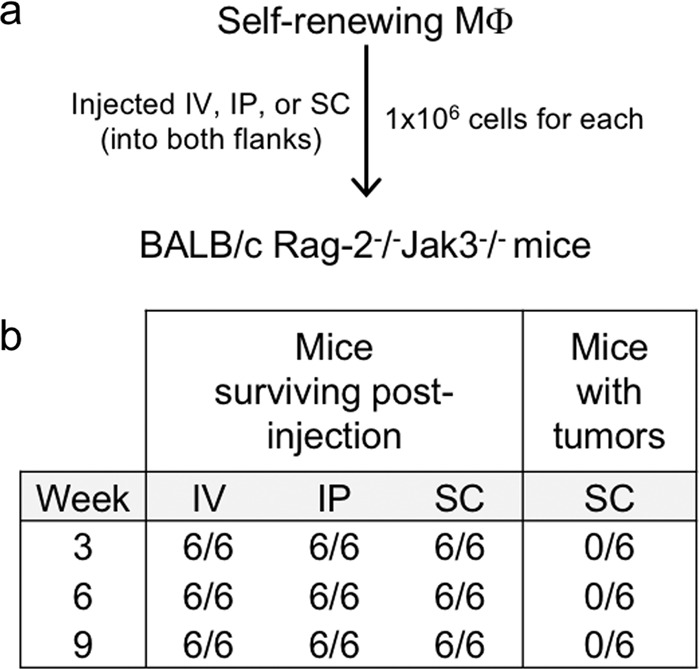
Non-tumorigenicity of the self-renewing macrophages. **a** Experimental outline for Fig. 2. MΦ macrophages, IV intravenously, IP intraperitoneally, SC subcutaneously. **b** BALB/c Rag-2^−/−^Jak3^−/−^ mice were injected with self-renewing macrophages (1 × 10^5^ cells/mouse, *n* = 6 for each group) intravenously (IV), intraperitoneally (IP), or subcutaneously (SC; into both flanks). The number of living mice and tumor-bearing mice (for SC group) was monitored at 3, 6, and 9 weeks after injection of cells.

**Fig. 3:**
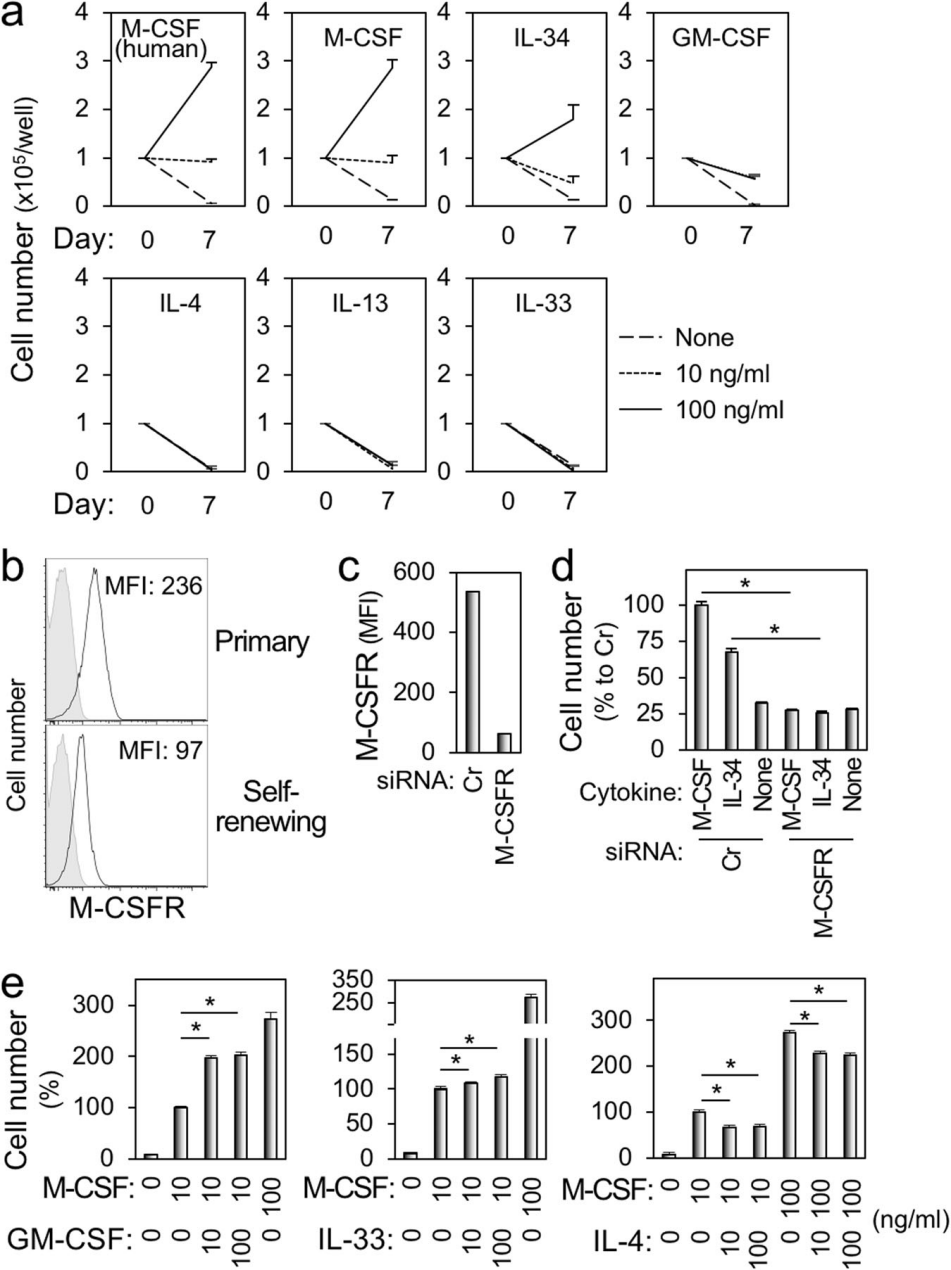
The proliferative response of the self-renewing macrophages to cytokines. **a** The self-renewing macrophages were cultured in the absence or presence of cytokine indicated (10 or 100 ng/ml) for 7 days. Regarding M-CSF, both human and mouse M-CSF were used. The number of viable cells was counted (mean ± SD, *n* = 3). **b**–**d** In **b**, the self-renewing macrophages (lower) and primary bone marrow-derived macrophages (upper) were analyzed for the expression of M-CSF receptor (M-CSFR) by flow cytometry. MFI mean fluorescence intensity. In **c**, the self-renewing macrophages were transfected with control (Cr) or M-CSF receptor-specific siRNA, cultured for 2 days in the presence of rhM-CSF, and subjected to flow cytometric analysis to verify the efficient knockdown. In **d**, the transfected cells prepared as in **c** were cultured for 2 days in the absence (none) or presence (100 ng/ml) of either rhM-CSF or rmIL-34, and their proliferation was assessed using the MTT assay. The number of cells is shown, by setting the value of the control (leftmost) as 100% (mean ± SD, *n* = 3), *p < 0.05. **e** The self-renewing macrophages were cultured for 2 days under the conditions indicated, and their proliferation was assessed using the MTT assay. rhM-CSF, rmGM-CSF, rmIL-33, and rmIL-4 were used. The number of cells is shown by setting the value of the control (M-CSF at 10 ng/ml, second left) as 100% (mean ± SD, *n* = 3), *p < 0.05.

The self-renewing macrophages did not give rise to tumors when injected into immunodeficient mice, irrespective of the injection route (Fig. 2, Supplementary Fig. 2), indicating that they were not tumorigenic. They required M-CSF or IL-34, but not IL-4, IL-13, IL-33, or GM-CSF, for the proliferation (Fig. 3a), and expressed M-CSF receptor (Fig. 3b), the common receptor for M-CSF and IL-34 (ref. ^19^). The knockdown of the receptor (Fig. 3c) abolished the M-CSF- or IL-34-dependent proliferation (Fig. 3d). When combined with M-CSF, IL-4 slightly inhibited the proliferation (Fig. 3e, right) without affecting M-CSF receptor expression (Supplementary Fig. 3). When combined with a suboptimal concentration of M-CSF (10 ng/ml), IL-33 slightly stimulated the proliferation (Fig. 3e, middle). Under the same conditions, GM-CSF strongly potentiated M-CSF-dependent proliferation (Fig. 3e, left), which was consistent with the fact that GM-CSF supported the survival of self-renewing macrophages (Fig. 3a). The self-renewing macrophages could be serially passaged in the presence of M-CSF at least for 1 year with a constant doubling time (data not shown). Thus, the self-renewing macrophages are non-tumorigenic and proliferated in the presence of M-CSF in unlimited numbers.

### Phenotypic properties of the self-renewing macrophages

**Fig. 4:**
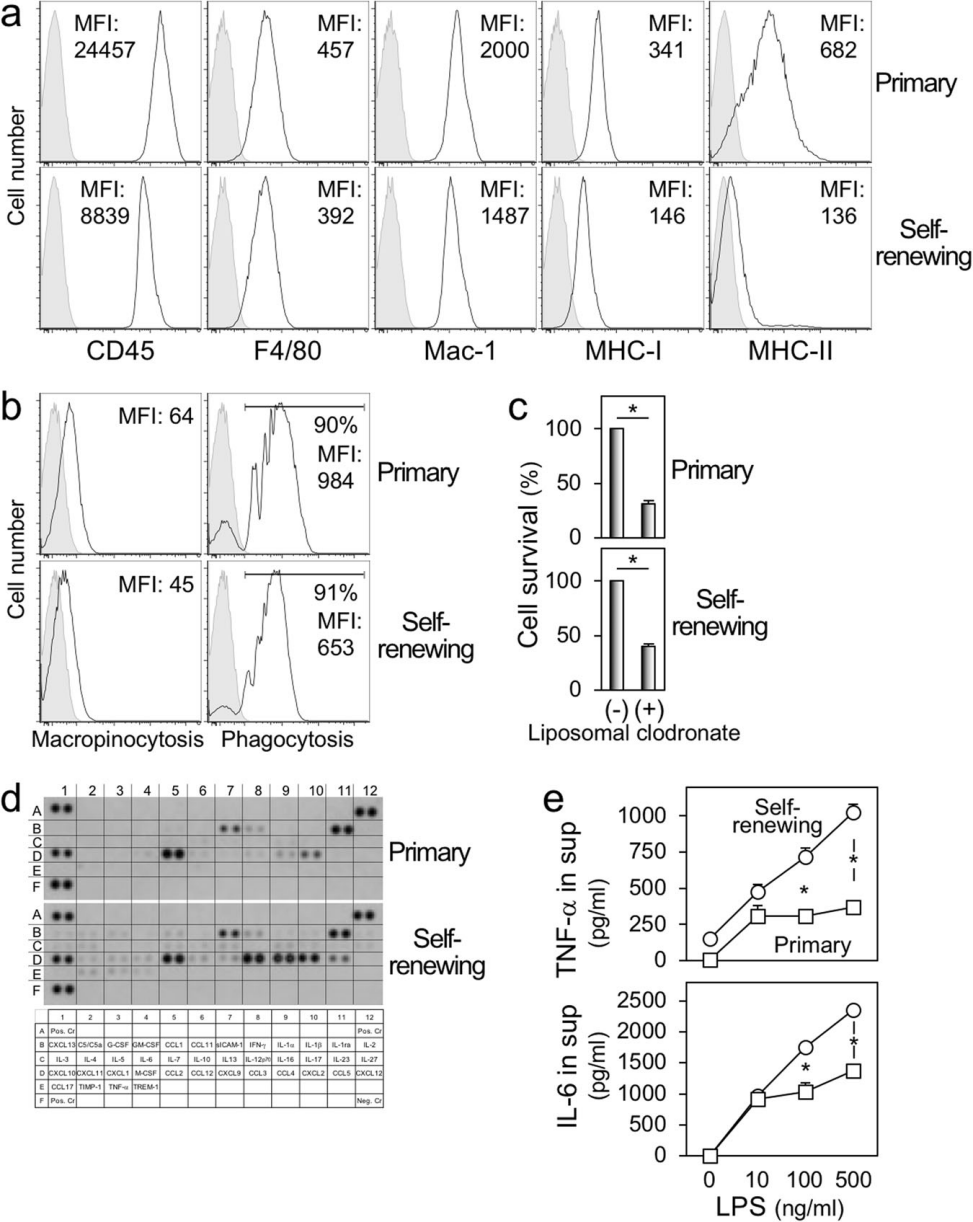
The phenotypes and functions of the self-renewing macrophages. **a**, **b** In **a**, the self-renewing macrophages (lower) and primary bone marrow-derived macrophages (upper) were analyzed for the expression of cell surface proteins indicated by flow cytometry. In **b**, they were analyzed for their macropinocytic (left) or phagocytic activities (right) by flow cytometry. In the right panels, the percentage of cells with phagocytic activities was also shown. MFI mean fluorescence intensity. **c** The self-renewing macrophages (lower) and primary bone marrow-derived macrophages (upper) were cultured in the presence of rhM-CSF alone or copresence with liposomal clodronate for 2 days. Their survival was assessed using the MTT assay. The number of cells is shown by setting the value of the control (liposomal clodronate free) as 100% (mean ± SD, *n* = 3), *p < 0.05. **d** The self-renewing macrophages (lower) and primary bone marrow-derived macrophages (upper) were analyzed for the relative levels of multiple cytokines and chemokines in their culture supernatants using an antibody array. **e** The self-renewing macrophages (circle) and primary bone marrow-derived macrophages (square) were cultured for 2 days in the presence of rhM-CSF and the indicated concentration of LPS. The concentration of TNF-α (upper) or IL-6 (lower) in the supernatants were quantified by ELISA (mean ± SD, *n* = 3). *p < 0.05.

**Fig. 5:**
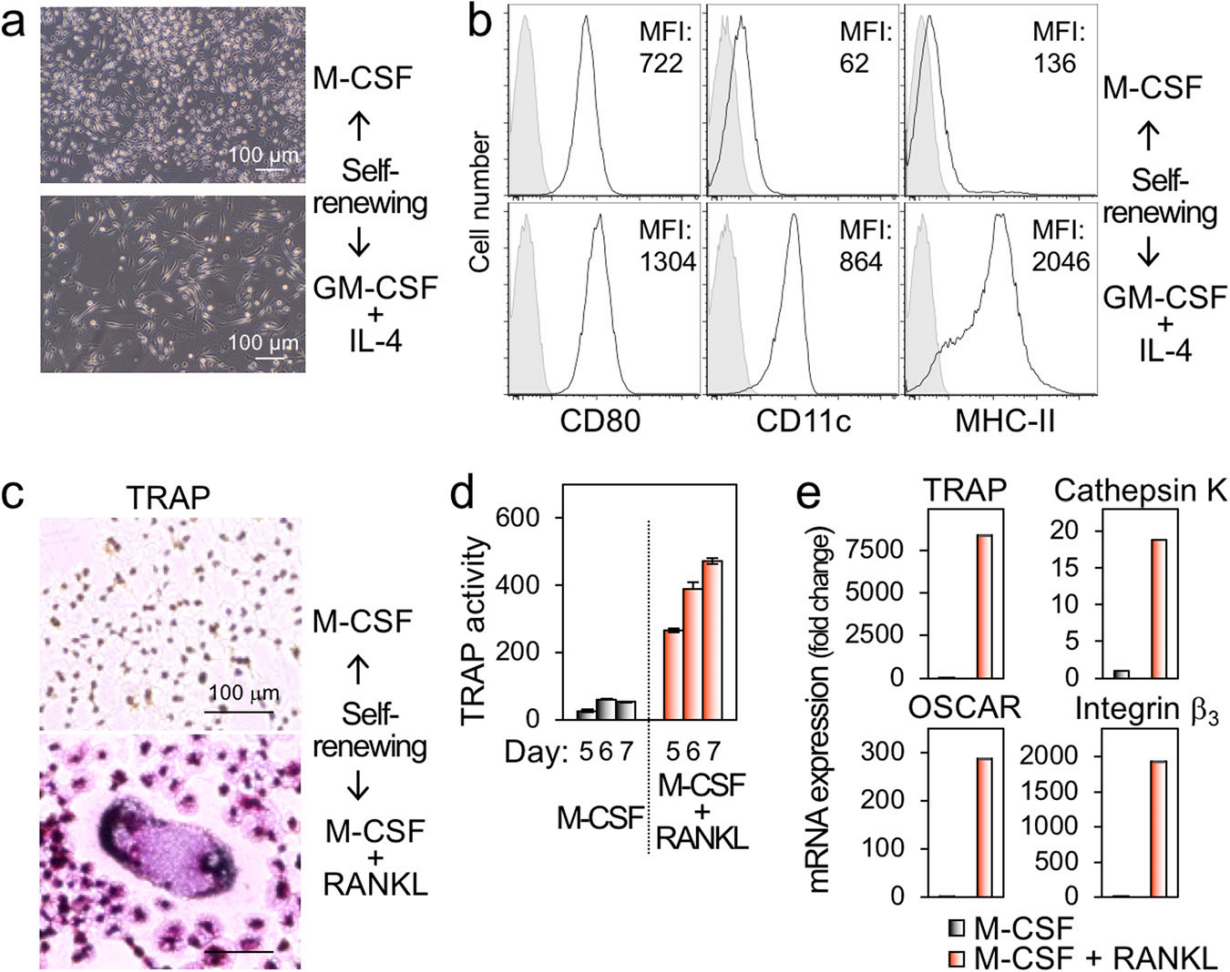
The differentiation of the self-renewing macrophages into dendritic cells or osteoclasts. **a** The self-renewing macrophages were cultured for 5 days with rhM-CSF (upper) alone or rmGM-CSF plus rmIL-4 (lower). **b** The self-renewing macrophages were cultured as in **a**, and analyzed for the expression of CD80, CD11c, or MHC-II by flow cytometry. MFI mean fluorescence intensity. **c** The self-renewing macrophages were cultured for 7 days with rhM-CSF (upper) alone or rhM-CSF plus rhRANKL (lower), and subjected to tartrate-resistant acid phosphatase (TRAP) staining. **d** The self-renewing macrophages were cultured as in **c** for 5, 6, or 7 days. Their TRAP activity was also quantified using the TRAP solution kit, and calculated as nmoles of p-NP per ml (mean ± SD, *n* = 3). **e** The self-renewing macrophages were cultured as in **c** for 3 days, and analyzed for the expression of the indicated genes by qRT-PCR.

We further analyzed the phenotypes of the self-renewing macrophages. They were positive for CD45 (hematopoietic cell marker), F4/80 and Mac-1 (macrophage marker), and MHC-I (Fig. 4a), although they minimally expressed MHC-II (Fig. 4a), as mentioned (Fig. 1e–g). There were genes expressed differently between self-renewing and primary macrophages (Supplementary Fig. 4a, b), but they had both macropinocytic and phagocytic activities (Fig. 4b), the hallmark functions of macrophages. Liposomal clodronate, which is widely used to deplete macrophages because macrophages efficiently ingest it via their phagocytic activity, reduced the number of self-renewing macrophages (Fig. 4c). There were differences in the production of several chemokines, including CCL3, CCL4, and CCL5, all of which are CCR5 ligands, between self-renewing and primary macrophages (Fig. 4d). Of interest, TNF-α or IL-6 induced by LPS in the self-renewing macrophages was significantly higher than that in primary macrophages (Fig. 4e), which was consistent with the enriched expression of genes involved in signaling of TLR4 (Supplementary Fig. 3c). The self-renewing macrophages exhibited morphological and phenotypical features of dendritic cells (Fig. 5a, b) or osteoclasts (Fig. 5c–e) when cultured under differentiation-inducing conditions, that is, GM-CSF plus IL-4 for dendritic cells, or M-CSF plus RANKL for osteoclasts. Thus, despite several differences from primary non-proliferating macrophages, including the expression of MHC-II and response to LPS, the self-renewing macrophages retained many features of cells of the monocytic lineage, including the differentiation into dendritic cells or osteoclasts.

### Upregulation of KLF2 upon M-CSF stimulation in the self-renewing macrophages

We next examined the molecular basis for the proliferative capacity of macrophages established in this study. Macrophages of mice with combined deficiency for transcription factors, MafB and c-Maf, continuously proliferated in an ex vivo culture with M-CSF^16^. In the Maf-DKO macrophages, several genes among those involved in self-renewal/pluripotency for embryonic stem (ES) cells showed an elevated level of basal expression^17^. Among those genes, a concomitant elevated expression of transcription factors, KLF2, KLF4, and c-Myc, appears to enable the continuous proliferation of Maf-DKO macrophages^17^. However, in the self-renewing macrophages, most of those genes including KLF4 and c-Myc did not show any obvious (>5-fold) elevated expression (data not shown). KLF2 showed an elevated expression, but its extent was modest (Supplementary Fig. 5a). The level of c-Maf (data not shown) and MafB (Supplementary Fig. 5a) in the self-renewing macrophages was comparable to that of primary macrophages.

**Fig. 6:**
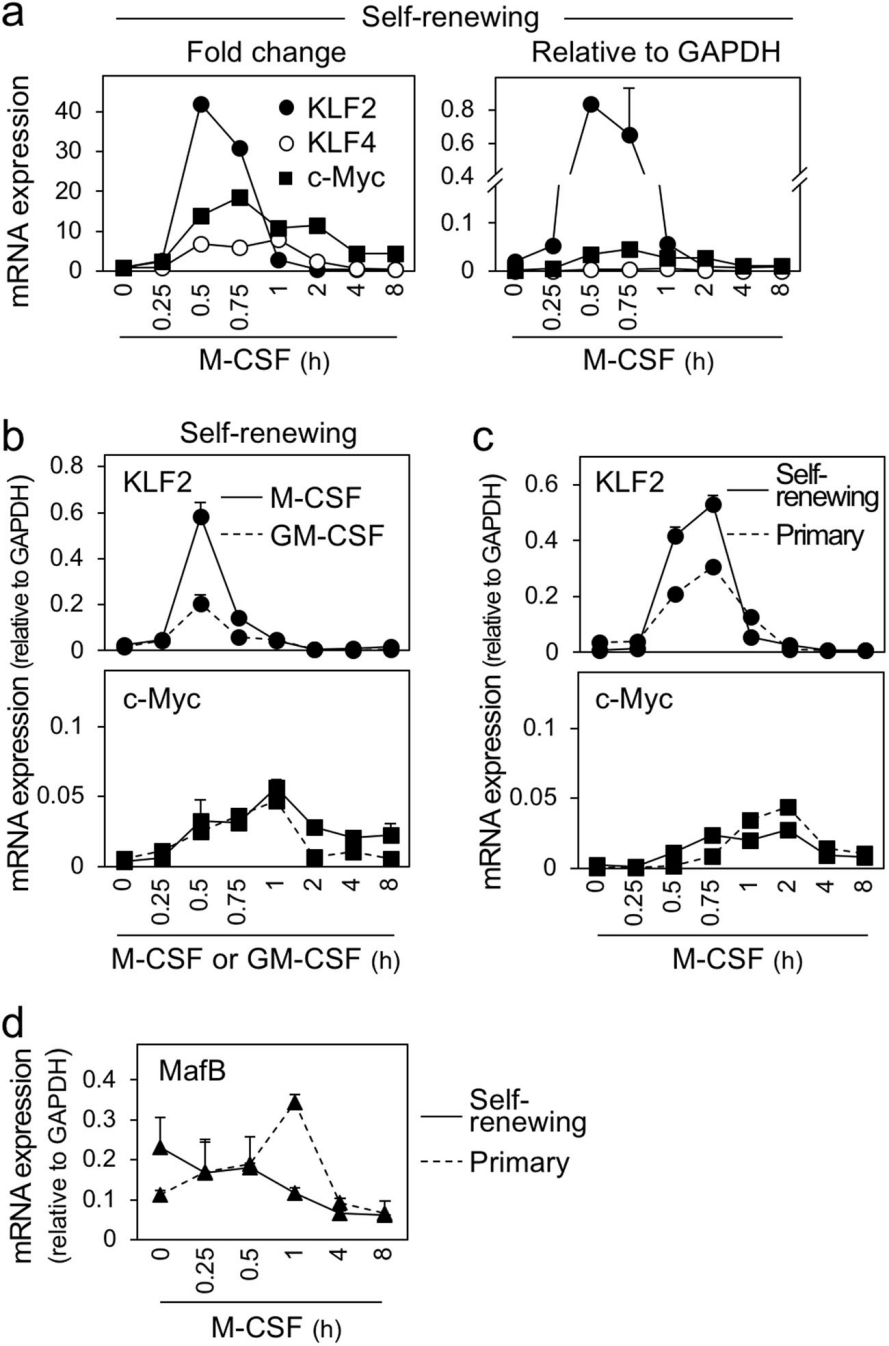
The upregulation of KLF2 upon M-CSF stimulation in the self-renewing macrophages. **a** The self-renewing macrophages were M-CSF-depleted and restimulated with rhM-CSF for the indicated periods, and analyzed for the expression of KLF2, KLF4, or c-Myc by qRT-PCR (mean ± SD, *n* = 3). The fold change for each gene relative to the unstimulated control and the expression level of these genes relative to GAPDH are shown in left and right, respectively. **b** The self-renewing macrophages were M-CSF-depleted and restimulated with rhM-CSF or rmGM-CSF for the indicated periods and analyzed for the expression of KLF2 (upper) or c-Myc (lower) by qRT-PCR (mean ± SD, *n* = 3). The expression level of these genes relative to GAPDH is shown. **c** The self-renewing macrophages or primary bone marrow-derived macrophages were M-CSF-depleted and restimulated with rhM-CSF for the indicated periods, and analyzed for the expression of KLF2 (upper) or c-Myc (lower) by qRT-PCR (mean ± SD, *n* = 3). The expression level of these genes relative to GAPDH is shown. **d** The self-renewing macrophages or primary bone marrow-derived macrophages were M-CSF-depleted and restimulated with rhM-CSF for the indicated periods, and analyzed for the expression of MafB by qRT-PCR (mean ± SD, *n* = 3). The expression level of these genes relative to GAPDH is shown.

Of importance, the self-renewing macrophages showed an upregulation of KLF2, KLF4, and c-Myc in response to M-CSF stimulation (Fig. 6a). KLF2 upregulation was most apparent in fold change (Fig. 6a, left), and its peak expression level was higher than the expression in ES cells (Supplementary Fig. 5b) and comparable to the expression of the housekeeping gene GAPDH (Fig. 6a, right). Furthermore, the KLF2 upregulation correlated with the proliferation of the self-renewing macrophages. (1) GM-CSF, which supported the survival, but not proliferation, of the self-renewing macrophages (see Fig. 3a), minimally upregulated KLF2, despite a comparable upregulation of c-Myc by these cytokines (Fig. 6b). (2) In the step of enrichment/expansion of the self-renewing macrophages, the cultures were composed of MHC-II^high^ and MHC-II^low^ fractions (see Fig. 1e–g). KLF2 upregulation by M-CSF stimulation in MHC-II^low^ fraction that contains self-renewing macrophages was more obvious than that in MHC-II^high^ fraction (data not shown). (3) The self-renewing macrophages showed a higher KLF2 upregulation than primary macrophages, despite a comparable upregulation of c-Myc in these cells (Fig. 6c).

Of interest, we also found that MafB, the suppressor of KLF2 expression^17^, was gradually downregulated after M-CSF stimulation in the self-renewing macrophages, which was in sharp contrast to its upregulation by the same stimulation in primary macrophages (Fig. 6d). In the same assay, we could not obtain reliable results for c-Maf because of its low basal level of expression (data not shown). Thus, the strong upregulation of KLF2 by M-CSF stimulation might be explained at least in part by the downregulation of MafB.

### Reduced proliferation and cell cycle arrest of the self-renewing macrophages by KLF2 knockdown

**Fig. 7:**
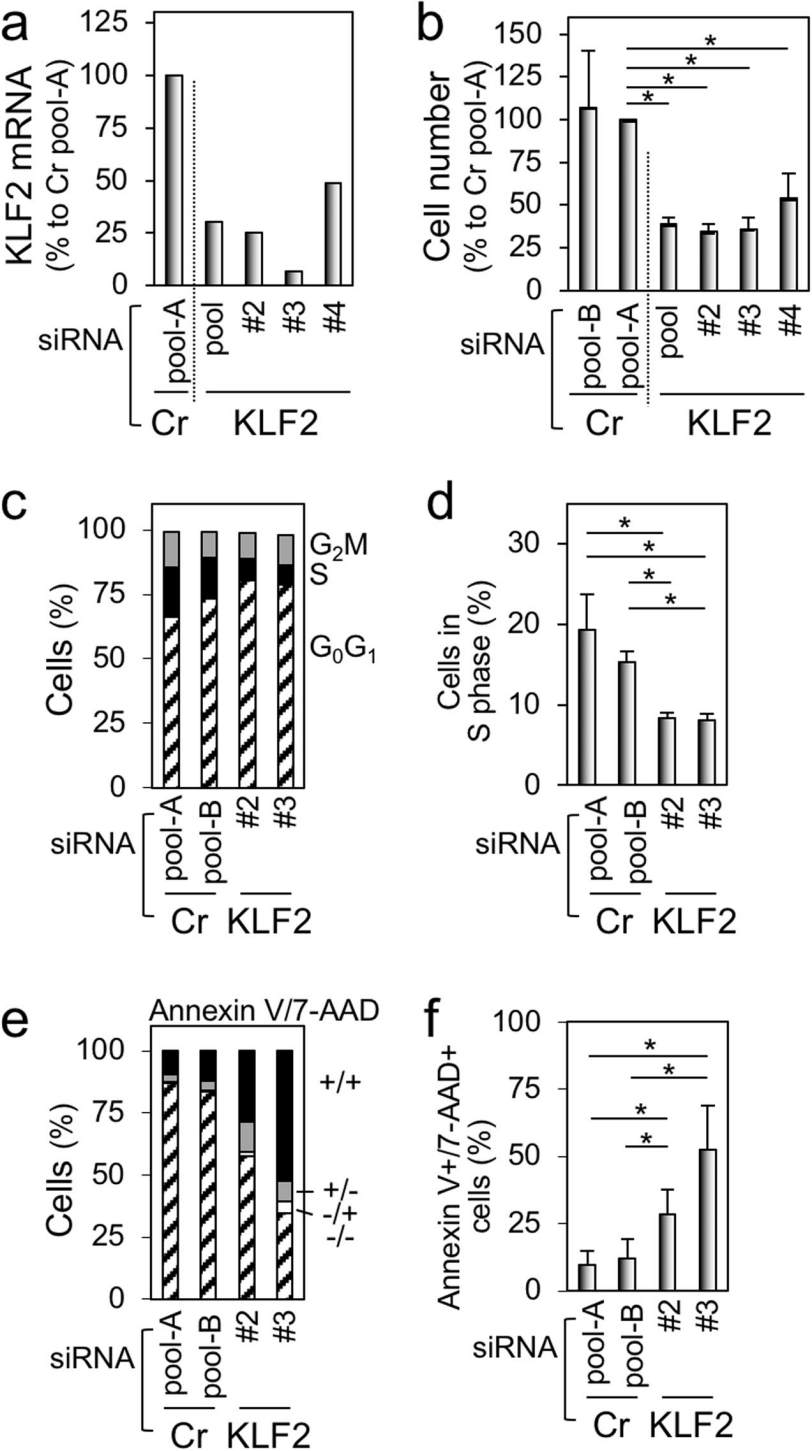
The effect of knockdown of KLF2 on proliferation of the self-renewing macrophages. **a** The self-renewing macrophages were transfected with siRNAs indicated, cultured for 2 days, and analyzed for the expression of KLF2 by qRT-PCR. The level of KLF2 is shown, by setting the value of the control (transfection with Cr pool-A siRNA) as 100%. **b** The self-renewing macrophages were transfected with siRNAs indicated, cultured for 2 days, and subjected to the MTT assay. The number of cells is shown, by setting the value of the control (transfection with Cr pool-A siRNA) as 100% (mean ± SD, *n* = 3), *p < 0.05. **c**, **d** The self-renewing macrophages were transfected with siRNAs indicated, cultured for 2 days, and subjected to the cell cycle analysis. In **d**, the percentage of cells in the S phase is summarized (mean ± SD, *n* = 3), *p < 0.05. **e**, **f** The self-renewing macrophages were transfected with siRNAs indicated, cultured for 2 days, and subjected to the apoptotic cell analysis. In **f**, the percentage of cells positive for both Annexin V and 7-AAD is summarized (mean ± SD, *n* = 3), *p < 0.05.

To test whether the upregulation of KLF2 is critical for the proliferation of the self-renewing macrophages, we performed knockdown experiments. A mixture (pool-A or pool-B) of four nontargeting siRNAs was used as a control. To knockdown KLF2, a mixture (pool) or individual siRNA (#2, #3, or #4) was used. When transfected, all the KLF2-specific siRNAs reduced the expression of KLF2 (Fig. 7a) and the number of the self-renewing macrophages (Fig. 7b), the latter of which was accompanied by the cell cycle arrest (Fig. 7c, d, Supplementary Fig. 6a) and increased Annexin V^+^/7-AAD^+^ cells (Fig. 7e, f, Supplementary Fig. 6b). Because we detected neither cleaved caspase 3 nor cleaved caspase 7 in the KLF2 knockdown culture (Supplemental Fig. 6c), the cell cycle arrest, but not apoptosis, might be the main reason for cell death induced by KLF2 knockdown.

**Fig. 8:**
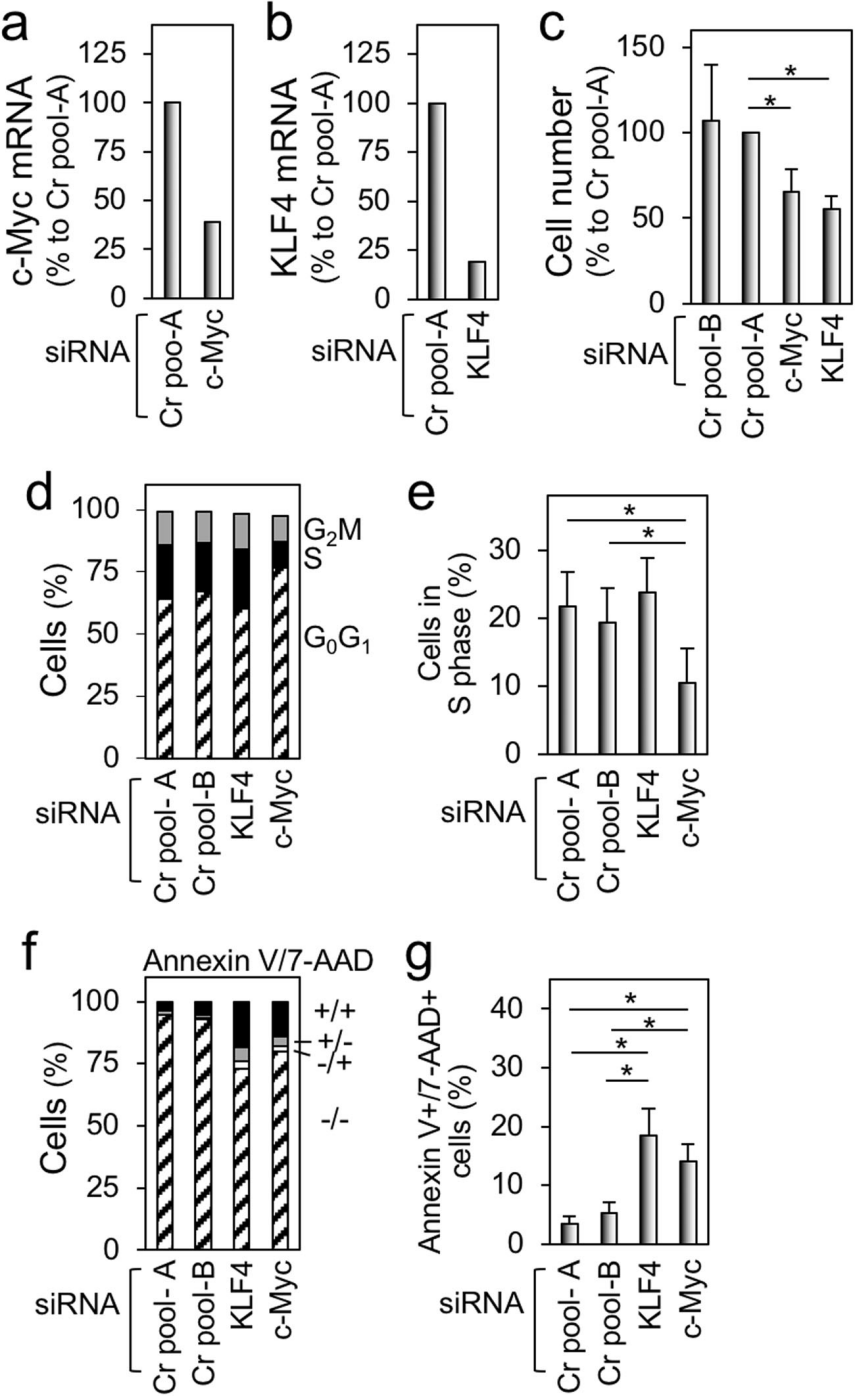
The effect of knockdown of c-Myc or KLF4 on the proliferation of the self-renewing macrophages. **a** The self-renewing macrophages were transfected with siRNAs indicated, cultured for 2 days, and analyzed for the expression of c-Myc by qRT-PCR. The level of c-Myc is shown, by setting the value of the control (transfection with Cr pool-A siRNA) as 100%. **b** The self-renewing macrophages were transfected with siRNAs indicated, cultured for 2 days, and analyzed for the expression of KLF4 by qRT-PCR. The level of c-Myc is shown, by setting the value of the control (transfection with Cr pool-A siRNA) as 100%. **c** The self-renewing macrophages were transfected with siRNAs indicated, cultured for 2 days, and subjected to the MTT assay. The number of cells is shown, by setting the value of the control (transfection with Cr pool-A siRNA) as 100% (mean ± SD, *n* = 3), *p < 0.05. **d**, **e** The self-renewing macrophages were transfected with siRNAs indicated, cultured for 2 days, and subjected to the cell cycle analysis. In **e**, the percentage of cells in the S phase is summarized (mean ± SD, *n* = 3), *p < 0.05. **f**, **g** The self-renewing macrophages were transfected with siRNAs indicated, cultured for 2 days, and subjected to the apoptotic cell analysis. In **g**, the percentage of cells positive for both Annexin V and 7-AAD is summarized (mean ± SD, *n* = 3), *p < 0.05.

As shown earlier (see Fig. 6a), c-Myc, the critical regulator of cell proliferation, was upregulated by M-CSF stimulation in the self-renewing macrophages whereas KLF4 showed a modest upregulation. Of interest, not only knockdown of c-Myc (Fig. 8a) but also knockdown of KLF4 (Fig. 8b) significantly reduced the number of the self-renewing macrophages (Fig. 8c). Although the cell cycle arrest was observed only by c-Myc knockdown (Fig. 8d, e, Supplementary Fig. 7a), the increased Annexin V^+^/7-AAD^+^ cells was induced by both c-Myc- and KLF4 knockdown (Fig. 8f, g, Supplementary Fig. 7b). These results might be explained by a network of self-renewal genes, as proposed in Maf-DKO macrophages^17^. Indeed, c-Myc knockdown did not affect the expression of KLF2 and KLF4 (Supplementary Fig. 8a), but KLF4 knockdown reduced the expression of KLF2 and c-Myc (Supplementary Fig. 8b). Thus, the reduced cell proliferation by KLF4 knockdown might be due to the reduced expression of KLF2 and/or c-Myc. Our results strongly suggest that the continuous proliferation of the self-renewing macrophages in the presence of M-CSF mainly requires upregulation of KLF2 and c-Myc.

## Discussion

In this study, we report the expansion of the adult bone marrow-derived, non-tumorigenic M-CSF-dependent self-renewing macrophages. This study supports the idea that homeostasis of tissue-resident macrophages occurs neither entirely by self-maintenance of embryo-derived cells nor renewal from the bone marrow, but depends on a combination of these processes. When combined with the precedent embryo-derived cell model^12^, our new cell model would be useful to study the biology of self-renewing macrophages with different origins.

Our cells resemble MPI cells, the mouse fetal liver-derived self-renewing macrophage model^12^, in weak expression of MHC-II (Fig. 4a) and high sensitivity to LPS (Fig. 4c). However, the in vitro doubling time of our cells (~4 days) is longer than that of the MPI cells^12^ and Maf-DKO macrophages^16^. We demonstrated the importance of KLF2 for the self-renewal of our cells (Figs. 6 and 7), but the role of KLF2 and other transcription factors, such as KLF4, c-Myc, c-Maf, or MafB, in the self-renewal of the MPI cells remains unclear. The dynamics of expression of these factors after mitogenic stimuli or the effect of knockdown of KLF2 or KLF4 on the proliferation has not been reported for the MPI cells. Of interest, at the basal level, the MPI cells exhibited higher expression of KLF4 and c-Myc (KLF2 was not included in the analysis) and lower expression of c-Maf and MafB, when compared to bone marrow-derived primary counterparts^12^. Such features are reminiscent of those of Maf-DKO macrophages^16,17^, and not seen in our cells (Supplementary Fig. 5a–c). Thus, this difference might explain the faster proliferation of the MPI cells. Also, the MPI cells and our cells are different in the responses to GM-CSF and M-CSF: the MPI cells proliferate more rapidly in the presence of GM-CSF^12^, whereas our cells proliferate in the presence of M-CSF, but not of GM-CSF (Fig. 3). These results may suggest that self-renewing macrophages with different origins have their proliferative capacity, and overlapping but not identical machinery for their proliferation.

M-CSF receptor ligands (M-CSF and IL-34), but not IL-4, IL-3, or IL-33 that has been reported to induce macrophage self-renewal in helminthiasis^20,21^, support the continuous proliferation of our cells. Consistent with this, M-CSF downregulates MafB in our cells, but not in primary counterparts (Fig. 6d). Although the underlying molecular mechanism remains unanswered, the downregulation of MafB may allow for the strong upregulation of KLF2 in our cells (Fig. 6a) because MafB is the suppressor of KLF2 expression^17^. Our finding is consistent with a study demonstrating that neuropeptide FF not only enhances the rate of proliferation of adipose tissue macrophages, but also downregulates MafB expression in the cells^22^. Recently, additional factors such as deacetylase Sirtuin1 (ref. ^23^), and transcription factors such as GATA6 (ref. ^24^), Bhlhe40 (refs. ^25,26^), and Bhlhe41 (ref. ^26^) have been reported to contribute to self-renewal of macrophages. Of these, Bhlhe40 has been shown to repress the expression of MafB and c-Maf^25^. Therefore, to clarify the precise molecular mechanism for M-CSF-dependent continuous proliferation of our cells, it will be important to include these new factors in the analysis.

Although the precursor cells for our cells and its frequency in the bone marrow remain unexplored, they can be expanded readily (Fig. 1) and independently of mouse strains (Supplementary Fig. 1). In mice studies, non-proliferating macrophages prepared by culturing bone marrow cells with M-CSF or GM-CSF for 5–7 days are generally used. Experiments with our cells skip the differentiation step and reduce the repeated use of mice. Our cells can also be utilized for the study of osteoclastogenesis (Fig. 5c), and would be useful for a wide range of fields, including host–pathogen interactions because the proliferation of host cells is critical for the mutual interaction. It would also be interesting to examine how our cells differentiate into tissue-specific macrophages, and how transcription factors that maintain the tissue-specific identities of macrophages, such as ZEB2 (refs. ^27–29^), are involved in the processes. In conclusion, our study shows the presence of self-renewing macrophages in the bone marrow, and provides the macrophage model that requires M-CSF and KLF2 as an extrinsic and intrinsic factor, respectively, for its unlimited proliferative capacity.

## Materials and methods

### Mice

C57BL/6 mice were used for the establishment of self-renewing macrophages. BALB/c mice were also used in selected experiments. The immunodeficient BALB/c Rag-2^−/−^Jak3^−/−^ mice^30^ were used for in vivo tumorigenic assay. These mice were maintained under specific pathogen-free conditions at the animal research facility of Kumamoto University. Procedures and protocols for animal experiments were approved by the institutional animal care and use committee of Kumamoto University.

### Cytokines

Recombinant human (rh) M-CSF (a gift from Morinaga Milk Industry, Kanagawa, Japan), which is cross-reactive to murine cells^31^, was used to expand and maintain self-renewing macrophages. Recombinant murine (rm) M-CSF (BioLegend) was also used in a selected experiment. Other cytokines used were as follows: rmGM-CSF (BioLegend), rmIL-4 (BioLegend), rmIL-13 (Peprotech), rmIL-33 (Peprotech), rmIL-34 (BioLegend), and rhRANKL (Oriental Yeast, Tokyo, Japan).

### Preparation of bone marrow monocyte-derived primary macrophages

Total bone marrow cells, which were obtained from C57BL/6 mice, were suspended into RPMI1640 media supplemented with 10% heat-inactivated FCS and 100 ng/ml rhM-CSF (complete media) at a density of 3 × 10^5^ cells/ml, seeded onto non-coated 10-cm dishes, and cultured for 5–7 days. The adherent cells were hereafter referred to as bone marrow monocyte-derived primary macrophages^32^.

### Establishment of bone marrow-derived self-renewing macrophages

Cells in the culture mentioned above were subjected to repeated detachment/reseeding: the adherent macrophages were detached using 0.25% trypsin and combined with floating cells, and they were suspended into fresh complete media at a density of 3 × 10^5^ cells/ml, seeded onto 10 cm non-coated dishes, and cultured for 5–7 days. In the fourth passage, most cells ceased to proliferate and begun to die. Instead, several growing colonies composed of macrophage-like adherent cells appeared. These cells were hereafter referred to as bone marrow-derived self-renewing macrophages, and maintained with complete media. Unless otherwise stated, bulk cells, but not clones, were used.

### Cell sorting

Cells in the culture mentioned above (the fourth passage) were detached from dishes using an enzyme-free cell dissociation buffer (Gibco) and stained with allophycocyanin (APC)-labeled anti-MHC-II (I-A/I-E) antibody (M5/114; BioLegend). MHC-II-low or MHC-II-high cell fraction in the live cell gate was sorted using a FACS Aria II (BD Biosciences). Sorted cells were subjected to proliferation assay.

### Flow cytometry for the expression of cell surface molecules, and phagocytic and macropinocytic activities

The expression of cell surface molecules on macrophages, which were detached from dishes using the enzyme-free cell dissociation buffer, was determined by flow cytometry on a FACSVerse (BD Biosciences) using FlowJo software (Tree Star). The following antibodies were used were: APC-anti-MHC-II (M5/114; BioLegend), FITC-anti-MHC-I (H-2) (M1/42; BioLegend), PE-anti-M-CSF receptor (AFS98; eBioscience), APC-anti-CD45 (30-F11; BioLegend), FITC-anti-F4/80 (BM8; BioLegend), FITC-anti-CD11b (Mac-1) (M1/70; BioLegend), FITC-anti-CD11c (N418; BioLegend), FITC-anti-CD80 (16-10A1; eBioscience), PE-anti-CCR2 (SA203G11; BioLegend), PE-anti-EphA2 (REA579; Miltenyi Biotec), and PE-anti-CD61 (integrin β_3_) (2C9.G2; BioLegend). The phagocytic activity of cells was determined by measuring the uptake of fluorescent microspheres (Fluoresbrite carboxylate microspheres, 0.7 μm in diameter; Polysciences), as described previously^33^. The macropinocytic activity was determined by measuring the uptake of Lucifer yellow (Sigma), as described previously^34^.

### In vitro cell proliferation assay and in vivo tumorigenic assay

The number of cells in cultures was assessed by the trypan blue dye exclusion method or using MTT reagent. In the MTT assay, the absorbance of wells was measured at 595 nm using a microplate reader (Bio-Rad). In a selected experiment, liposomal clodronate (FormuMax Scientific) was added to cultures as described previously^35^. In the tumorigenic assay, BALB/c Rag-2^−/−^Jak3^−/−^ mice^30^ were injected with self-renewing macrophages (1 × 10^5^ cells per mouse and six mice for each group) intravenously, intraperitoneally, or subcutaneously (into both flanks). The number of living and tumor-free mice was monitored at 3, 6, and 9 weeks after injection of cells.

### Cytokine/chemokine production

The relative levels of various cytokines and chemokines in media conditioned by macrophages were analyzed using a mouse cytokine array (R&D Systems) according to the manufacturer’s instructions. The concentrations of TNF-α and IL-6 in media conditions by macrophages stimulated with LPS (from *Escherichia coli*, serotype 0111:B4; Alexis) were determined using ELISA kits. Kits used were as follows: ELISA MAX standard set for IL-6 (BioLegend) and Quantikine kit for TNF-α (R&D Systems).

### Microarray

Total RNA was prepared from macrophages, and microarray analysis was performed at TaKaRa-Bio (Shiga, Japan) using SurePrint G3 Mouse Gene Expression 8x60K v2 and GeneSpring 12.5 software (Agilent Technologies). Microarray data have been deposited in the National Center for Biotechnology Information Gene Expression Omnibus (GSE142619).

### Dendritic cell differentiation assay

Self-renewing macrophages were cultured with RPMI1640 medium supplemented with 10% FCS, 10 ng/ml rmGM-CSF, and 10 ng/ml rmIL-4 for 5 days. Cells were detached from dishes using the enzyme-free cell dissociation buffer, and analyzed for the expression of CD11c, CD80, and MHC-II by the flow cytometry.

### Osteoclast differentiation assay

Self-renewing macrophages were cultured with α-MEM medium supplemented with 10% FCS, 100 ng/ml rhM-CSF, and 100 ng/ml rhRANKL for up to 7 days. Cells were fixed and subjected to tartrate-resistant acid phosphatase (TRAP) staining as described previously^36^. TRAP activity was also quantified using the TRAP solution kit (Oriental Yeast). qRT-PCR for the expression of TRAP, cathepsin K, OSCAR, or integrin β_3_ was performed as described previously^36^.

### Real-time RT-PCR

Macrophages were analyzed for the expression of KLF2, KLF4, c-Myc, c-Maf, MafB, or PU.1 mRNA followed by the normalization to the mRNA level of GAPDH, as described previously^37^. In brief, RNA was isolated using the ISOGEN II reagent (Nippon Gene, Tokyo, Japan), and each cDNA was prepared using M-MLV RT (Invitrogen). qPCR was performed with SYBR Premix Ex Taq II (TaKaRa-Bio) using a LightCycler (Roche). In a selected experiment, mouse ES cells^38^ were used as a reference. To assess the levels of mRNA of target genes after the stimulation with M-CSF macrophages maintained with rhM-CSF were factor-depleted for 6 h or longer^39^, and then left untreated or treated with 100 ng/ml rhM-CSF. In selected experiments, rhGM-CSF (10 ng/ml) was used as a reference. RNA isolated from these treated cells was also subjected to real-time RT-PCR. Primer sequences are as follows: 5′-accaagagctcgcacctaaa-3′ and 5′-gtggcactgaaagggtctgt-3′ (KLF2), 5′-ctgaacagcagggactgtca-3′ and 5′-gtgtgggtggctgttctttt-3′ (KLF4), 5′-cggacacacaacgtcttggaa-3′ and 5′-aggatgtaggcggtggctttt-3′ (c-Myc), 5′-gtgagaaaagcagttgcccg-3′ and 5′-agcttctcaccataccatcgac-3′ (c-Maf), 5′-tgaatttgctggcactgctg-3′ and 5′-aagcaccatgcggttcataca-3′ (MafB), 5′-gtagcgcaagagatttatgcaaac-3′ and 5′-gcacaagttcctgattttatcgaa-3′ (PU.1), and 5′-aaggtcatcccagagctgaa-3′ and 5′-ctgcttcaccaccttcttga-3′ (GAPDH). To show the fold change for each target mRNA, its level was normalized to GAPDH level in the same sample. To clarify an abundance of each target mRNA, its level was also shown by setting GAPDH level as 1.

### RNA interference

Knockdown of target genes (M-CSF receptor, KLF2, KLF4, and c-Myc) in macrophages was performed using Lipofectamine RNAiMAX reagent (Invitrogen) and siRNA, as described previously^40^. In brief, cells were suspended in antibiotic-free RPMI1640/10% FCS/100 ng/ml rhM-CSF media, seeded onto six-well plates (2 × 10^5^ cells/well), and cultured for 1 day. Cells were then transfected with 50 pmol/well of siRNA using 7.5 μl/well of Lipofectamine RNAiMAX. After 6 h of transfection, media were replaced with fresh complete media containing antibiotics, and cells were cultured for another 2 days. The knockdown efficiency was assessed by qRT-PCR, and transfected cells were subjected to experiments, including proliferation assay, cell cycle analysis, and apoptotic cell analysis. siRNAs used are as follows (all from Dharmacon): nontargeting siRNAs (pool #1; D-001206-13, and pool #2; D-001206-14), M-CSF receptor-specific siRNA (pool; M-044650), KLF2-specific siRNAs (pool; M-046974, #2; M-046974-02, #3; M-046974-03, and #4; M-046974-04), KLF4-specific siRNA (pool; M-040001), and c-Myc-specific siRNA (pool; M-04813).

### Cell cycle analysis and apoptotic cell analysis

Self-renewing macrophages transfected with siRNA were detached from dishes using the enzyme-free cell dissociation buffer and fixed with 70% ethanol. The nuclei were stained with propidium iodide in RNase-containing buffer and analyzed on FACSVerse. Cell cycle (G_0_G_1_, G_2_M, and S) was analyzed using FlowJo software. Cells were also analyzed using the PE Annexin V apoptosis detection kit with 7-AAD (BioLegend) according to the manufacturer’s instructions.

### Statistical analysis

The statistical significance of the inter-sample differences was determined using the paired Student’s *t*-test. *P*-value < 0.05 was considered significant.

## Supplementary information

Supplemental Figure Legends

Supplemental Figure S1

Supplemental Figure S2

Supplemental Figure S3

Supplemental Figure S4

Supplemental Figure S5

Supplemental Figure S6

Supplemental Figure S7

Supplemental Figure S8
