# Supplementary material for: Establishment of bone marrow-derived M-CSF receptor-dependent self-renewing macrophages
Source: Cell Death Discov. 2020 Jul 23;6:63. doi: 10.1038/s41420-020-00300-3 (PMC7378060; doi:10.1038/s41420-020-00300-3)
Supplement: Supplementary file 1 — Supplemental Figure Legends [file 41420_2020_300_MOESM1_ESM.docx]

**Supplemental Figure Legends**

**Supplementary Fig. S1 The proliferation of self-renewing macrophage clones obtained from C57BL/6 or BALB/c mice.**

The clones were obtained as in Fig. 1a, and cultured in the presence of rhM-CSF as indicated. The number of viable cells was counted (mean±SD, n=3).

**Supplementary Fig. S2 Non-tumorigenicity of the self-renewing macrophages.**

BALB/c Rag-2^-/-^Jak3^-/-^ mice were injected with self-renewing macrophages (1 x 10^5^ cells/mouse, n=6) subcutaneously (SC; into both flanks) as in Fig. 2. These mice were monitored for the formation of tumors at 9 weeks after injection of cells.

**Supplementary Fig. S3 M-CSF receptor expression in IL-4-treated self-renewing macrophages.**

The self-renewing macrophages were cultured for 2 days with rhM-CSF alone or rhM-CSF plus rmIL-4 (10 or 100 ng/ml) as indicated, and analyzed for the expression of M-CSF receptor (M-CSFR) by flow cytometry. MFI, mean fluorescence intensity.

**Supplementary Fig. S4 Gene expression profile of the self-renewing macrophages.**

(**a**) Microarray analysis showing genes with differential expression between the self-renewing macrophages and primary bone marrow-derived macrophages.

(**b**) In the microarray analysis, Eph2 was high in the self-renewing macrophages whereas CD61 was high in primary bone marrow-derived macrophages (data not shown), which was confirmed by flow cytometry.

(**c**) Enrichment plots of activated TLR4 signaling (upper) and NF-κB/MAPK activation mediated by TLR4 signaling (lower) following Gene set enrichment analysis (GSEA) of the microarray data from self-renewing macrophages versus primary bone marrow-derived macrophages.

**Supplementary Fig. S5 The expression of transcription factors in the self-renewing macrophages.**

(**a**) Primary bone marrow-derived macrophages (Primary), once passaged primary bone marrow-derived macrophages (+1 passage), and the self-renewing macrophage clone (#2, #5 and #6) were analyzed for the expression of KLF2, KLF4, c-Myc or MafB by qRT-PCR (mean±SD, n=3). The fold change for each gene relative to primary bone marrow-derived macrophages is shown.

(**b**) ES cells and the self-renewing macrophages left untreated or stimulated with rhM-CSF for 30 minutes were analyzed for the expression of KLF2, KLF4 or c-Myc by qRT-PCR (mean±SD, n=3). The fold change for each gene relative to the un-stimulated self-renewing macrophages and the expression level of these genes relative to GAPDH are shown in upper and lower, respectively.

**Supplementary Fig. S6 The effect of knockdown of KLF2 on cell cycle and apoptosis of the self-renewing macrophages.**

(**a**, **b**) The self-renewing macrophages were transfected with siRNAs indicated, cultured for 2 days, and subjected to the cell cycle analysis (**a**) or apoptotic cell analysis (**b**). Typical examples are shown. (**c**) The self-renewing macrophages were transfected with siRNAs indicated, cultured for 2 days, and subjected to western blotting using the following antibodies: anti-cleaved caspase 3 (#9661; Cell Signaling Technology), anti-cleaved caspase 7 (#8438; Cell Signaling Technology), and g-tubulin (C-20; Santa Cruz Biotechnology, as a loading control). The un-transfected self-renewing macrophages cultured with M-CSF (M-CSF+) or M-CSF-depleted for 12 hours (M-CSF-) were also analyzed as references. Western blotting was performed as described previously (Panaampon J, Kudo E, Kariya R, Okada S. Ephedrine enhances HIV-1 reactivation from latency through elevating tumor necrosis factor receptor II (TNFRII) expression. *Heliyon* 2019; **5**: e02490). Data shown are representative of 3 independent experiments with similar results.

**Supplementary Fig. S7 The effect of knockdown of c-Myc or KLF4 on cell cycle and apoptosis of the self-renewing macrophages.**

(**a**, **b**) The self-renewing macrophages were transfected with siRNAs indicated, cultured for 2 days, and subjected to the cell cycle analysis (**a**) or apoptotic cell analysis (**b**). Typical examples are shown.

**Supplementary Fig. S8 The effect of knockdown of c-Myc or KLF4 on the expression of other transcriptions factors in the self-renewing macrophages.**

(**a**, **b**) The self-renewing macrophages were transfected with siRNAs indicated, cultured for 2 days, and analyzed for the expression of transcription factors indicated by qRT-PCR. The macrophage lineage-defining transcription factor PU.1 is also included. The level of these transcription factors is shown by setting the value of the control (transfection with Cr pool-A siRNA) as 100% (mean±SD, n=3). **p*<0.05.
