# Supplementary figures and images for: Establishment of bone marrow-derived M-CSF receptor-dependent self-renewing macrophages
Source: Cell Death Discov. 2020 Jul 23;6:63. doi: 10.1038/s41420-020-00300-3 (PMC7378060; doi:10.1038/s41420-020-00300-3)

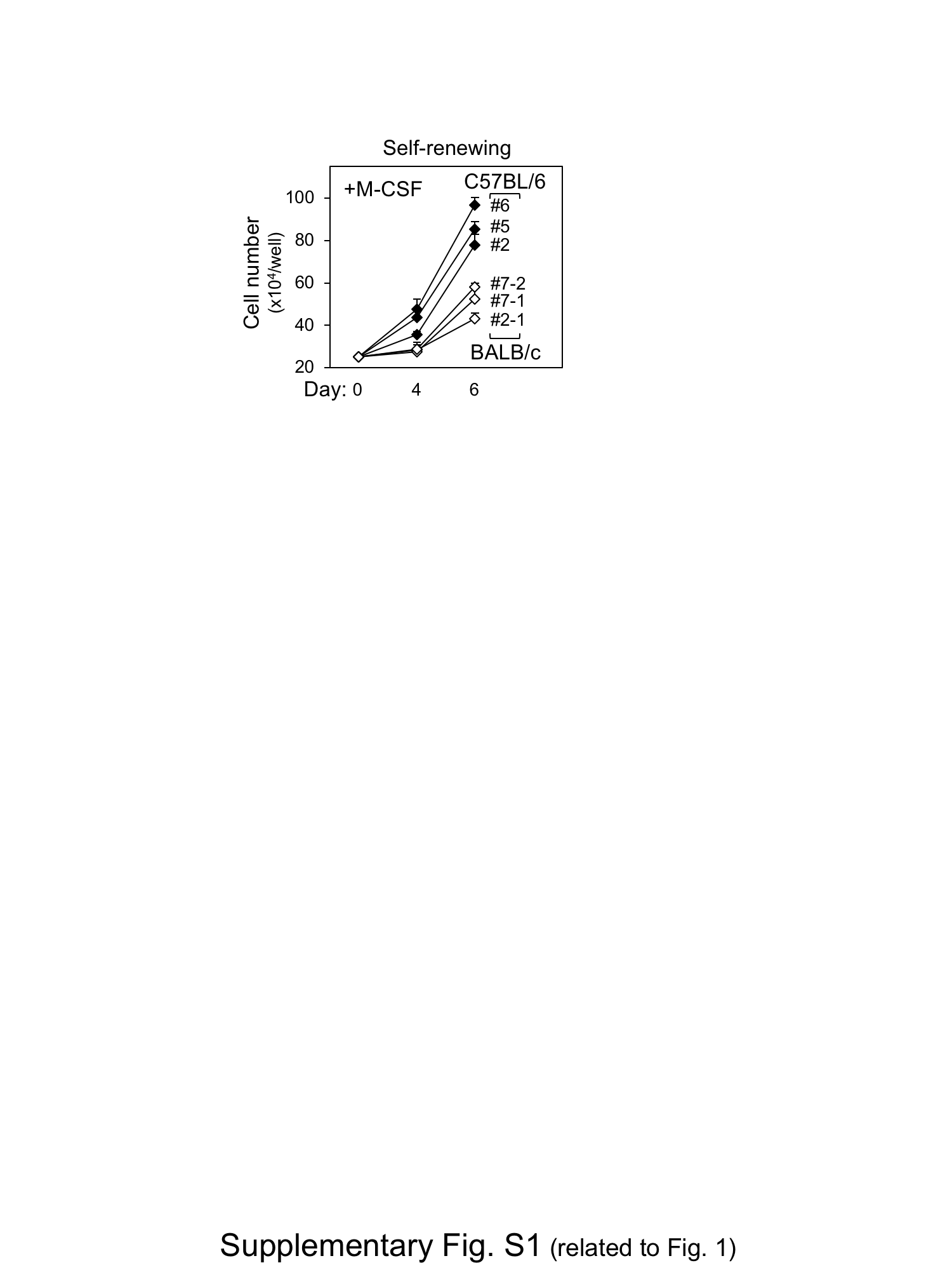

Supplement: Supplementary file 2 — Supplemental Figure S1 [file 41420_2020_300_MOESM2_ESM.png]

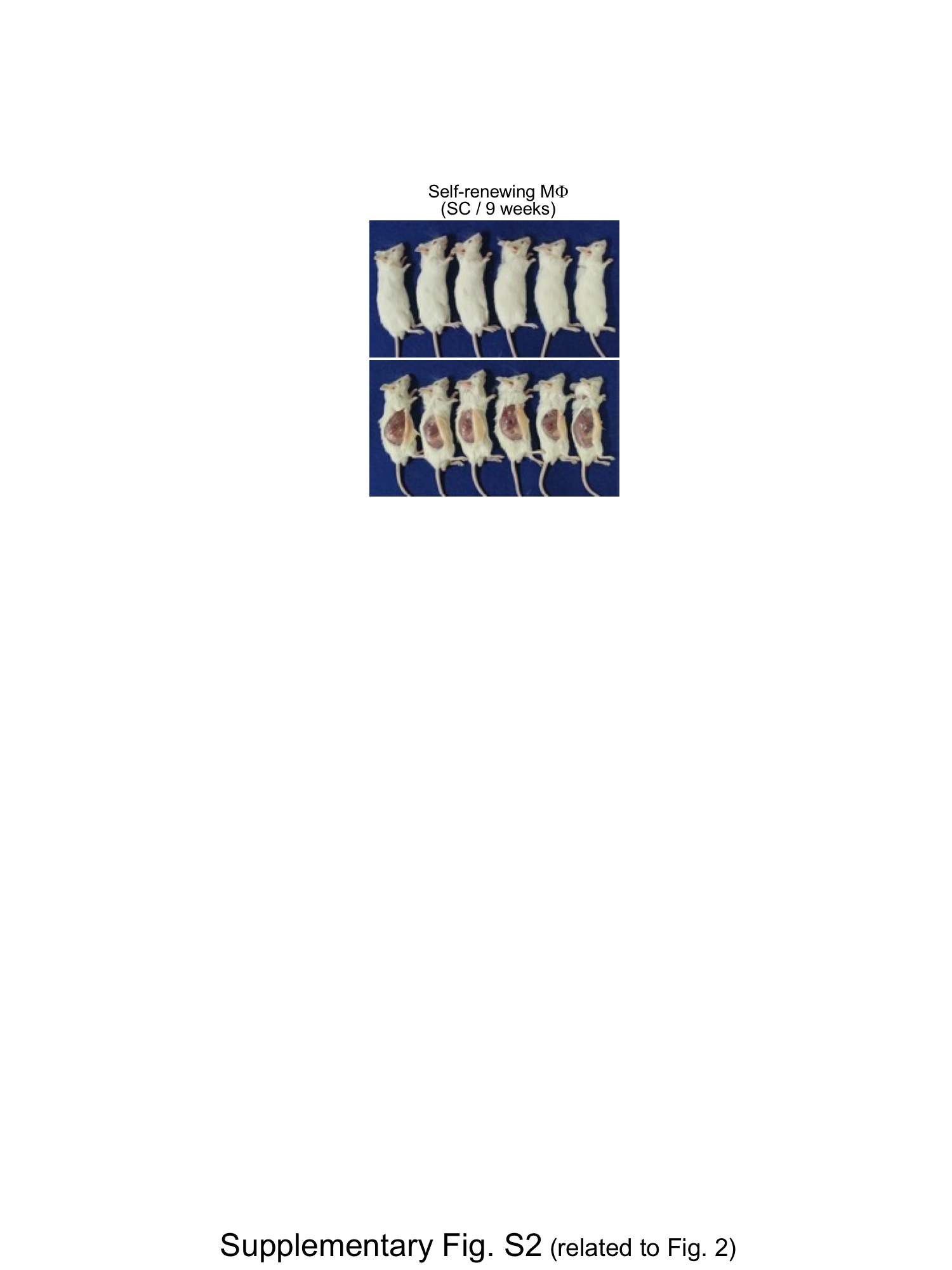

Supplement: Supplementary file 3 — Supplemental Figure S2 [file 41420_2020_300_MOESM3_ESM.png]

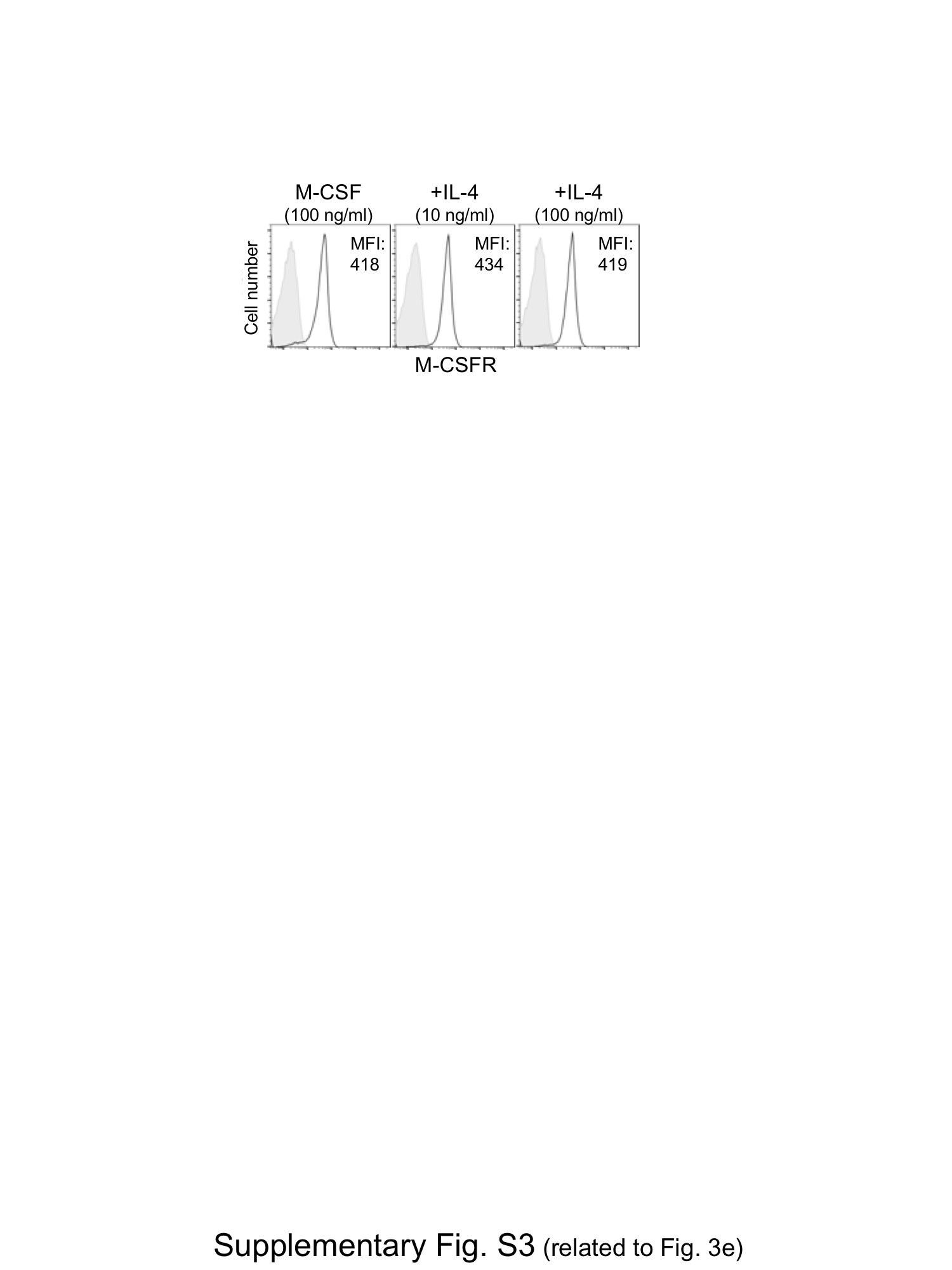

Supplement: Supplementary file 4 — Supplemental Figure S3 [file 41420_2020_300_MOESM4_ESM.png]

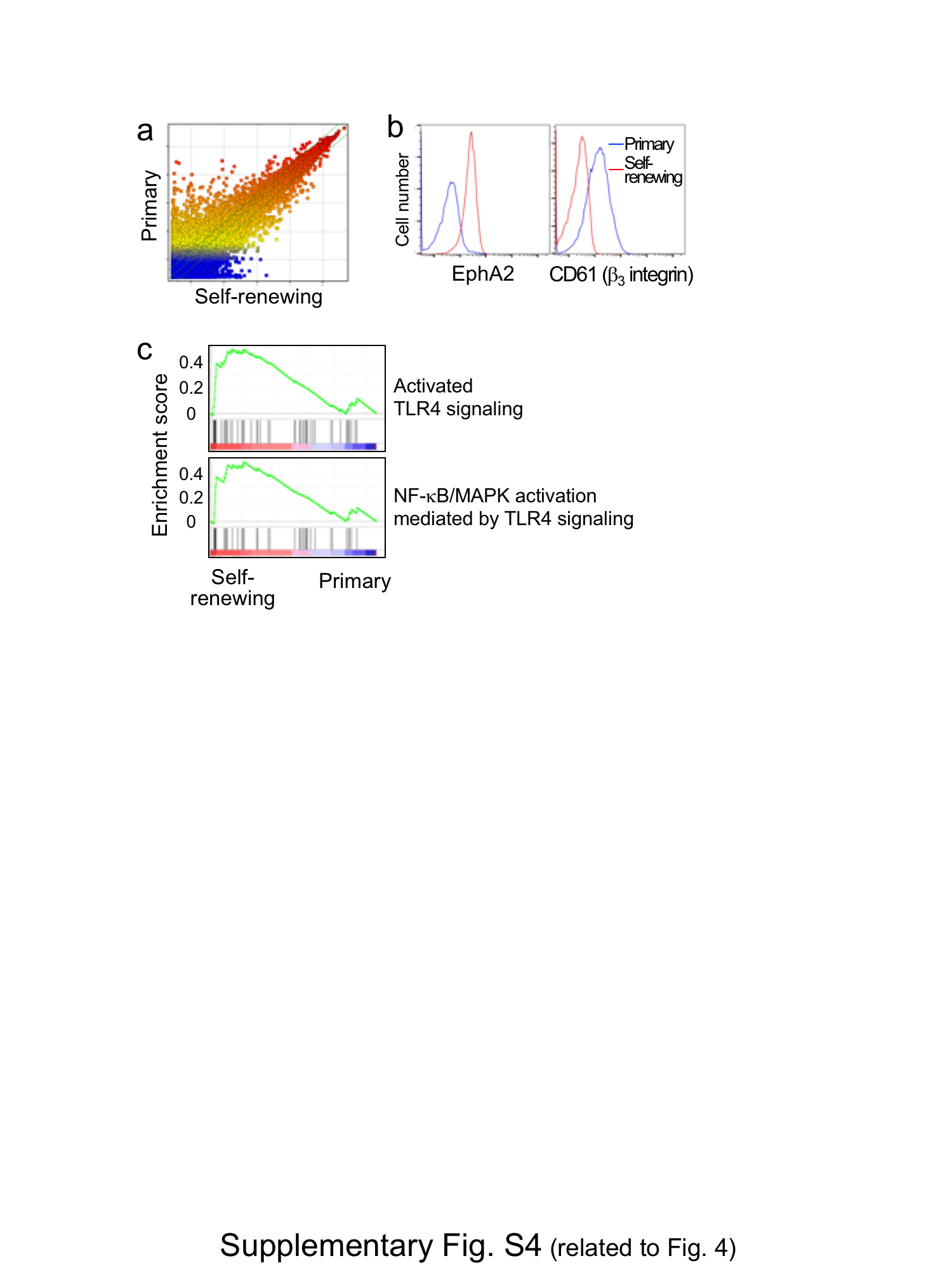

Supplement: Supplementary file 5 — Supplemental Figure S4 [file 41420_2020_300_MOESM5_ESM.png]

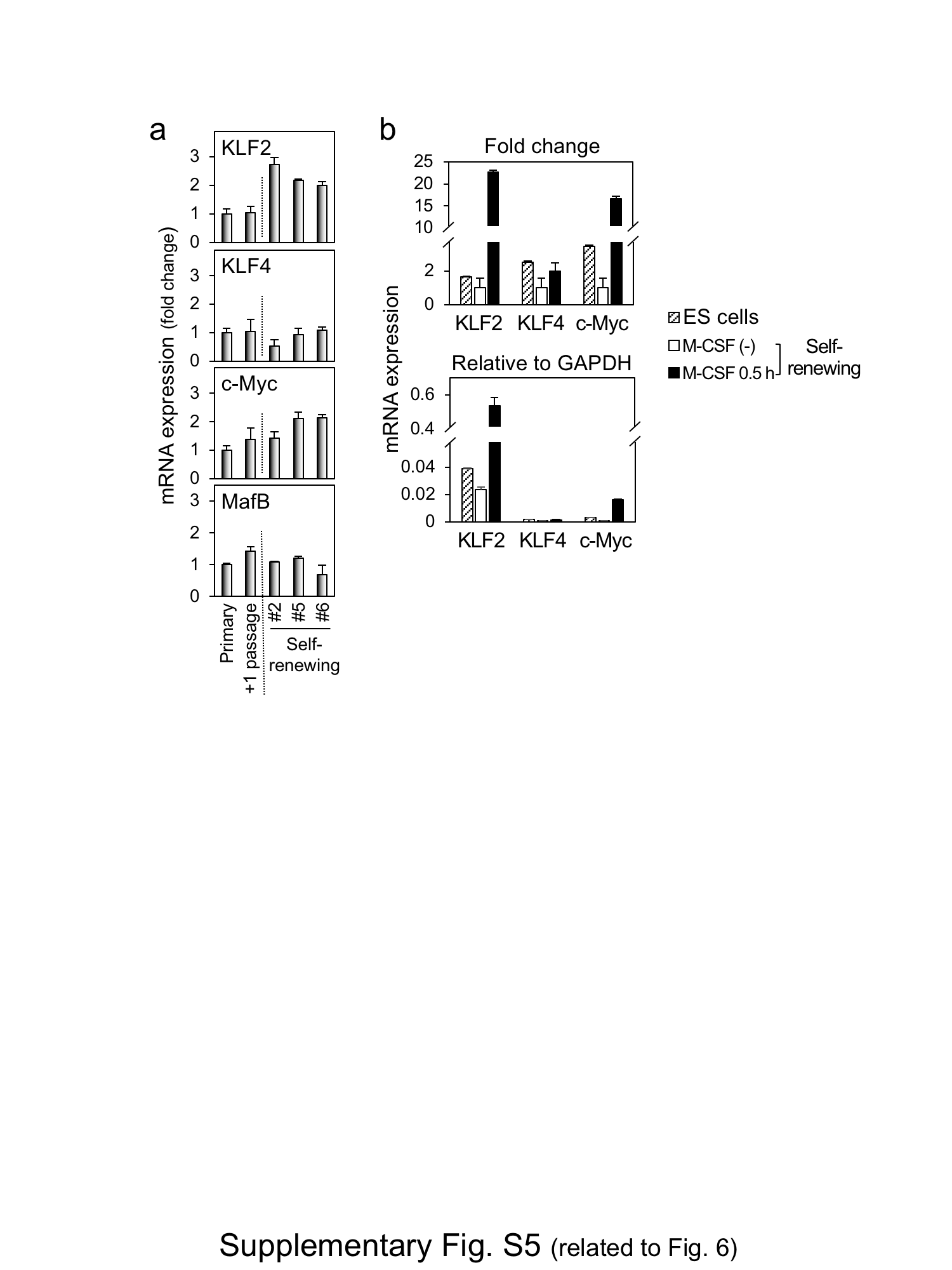

Supplement: Supplementary file 6 — Supplemental Figure S5 [file 41420_2020_300_MOESM6_ESM.png]

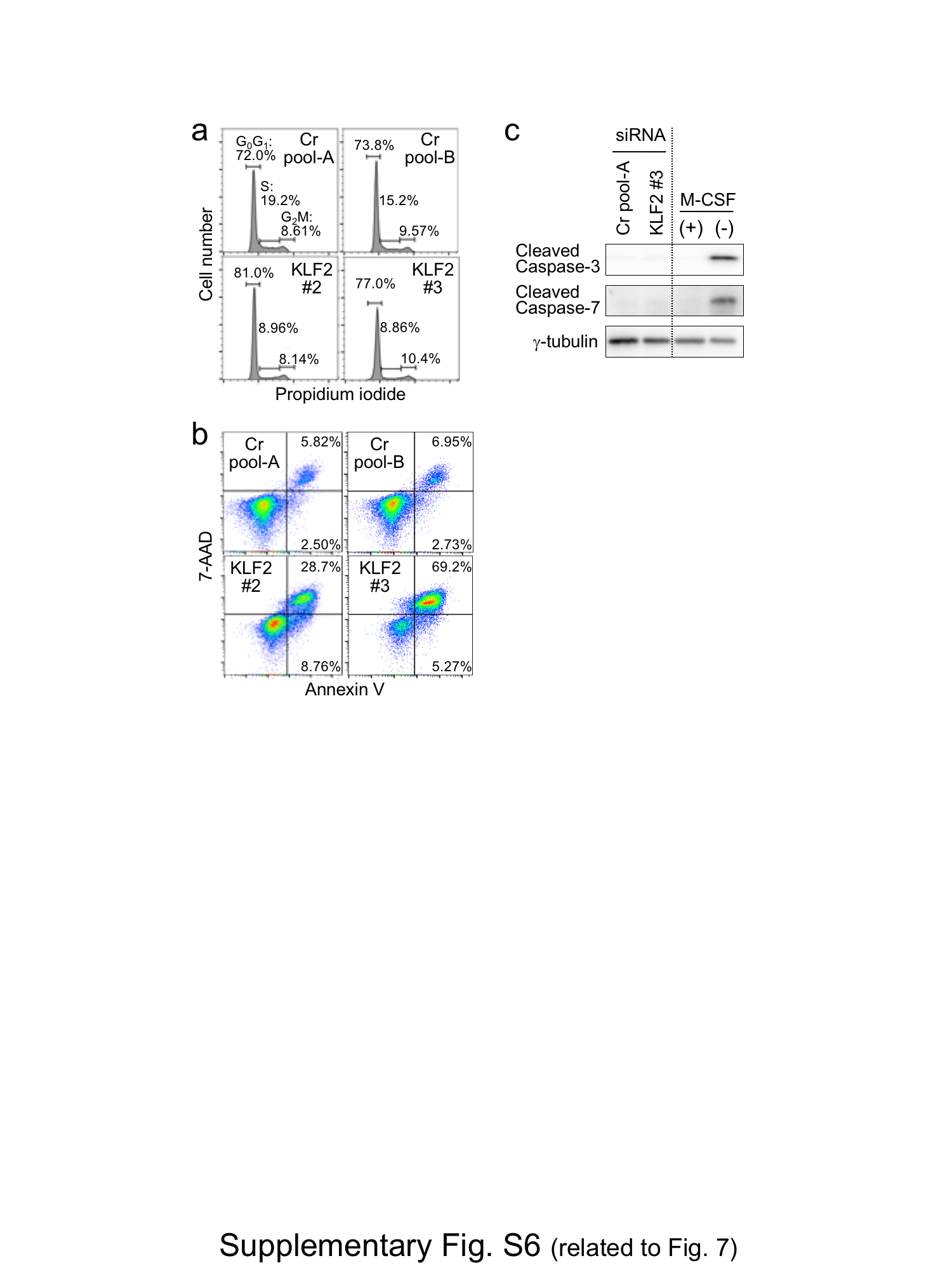

Supplement: Supplementary file 7 — Supplemental Figure S6 [file 41420_2020_300_MOESM7_ESM.png]

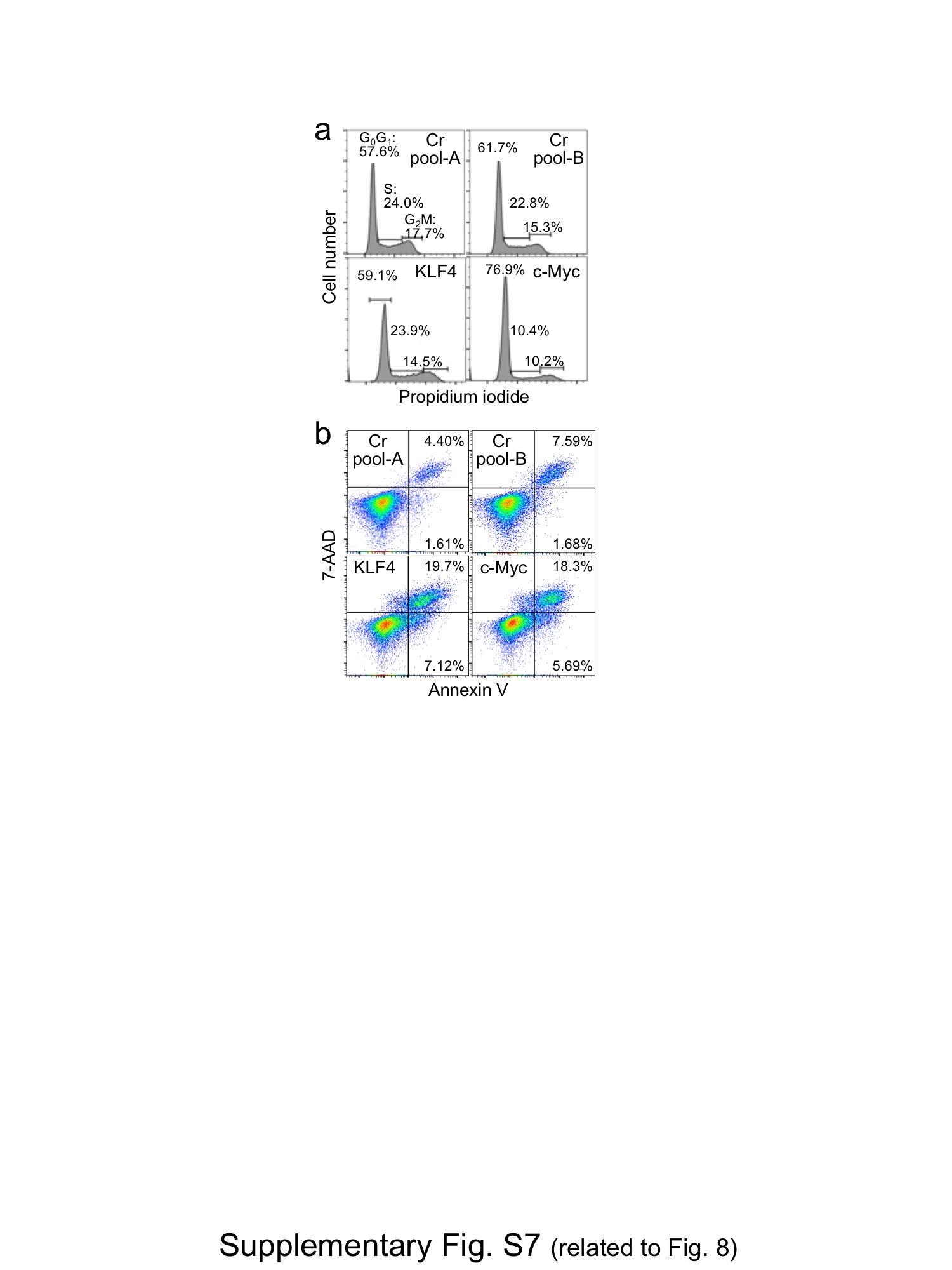

Supplement: Supplementary file 8 — Supplemental Figure S7 [file 41420_2020_300_MOESM8_ESM.png]

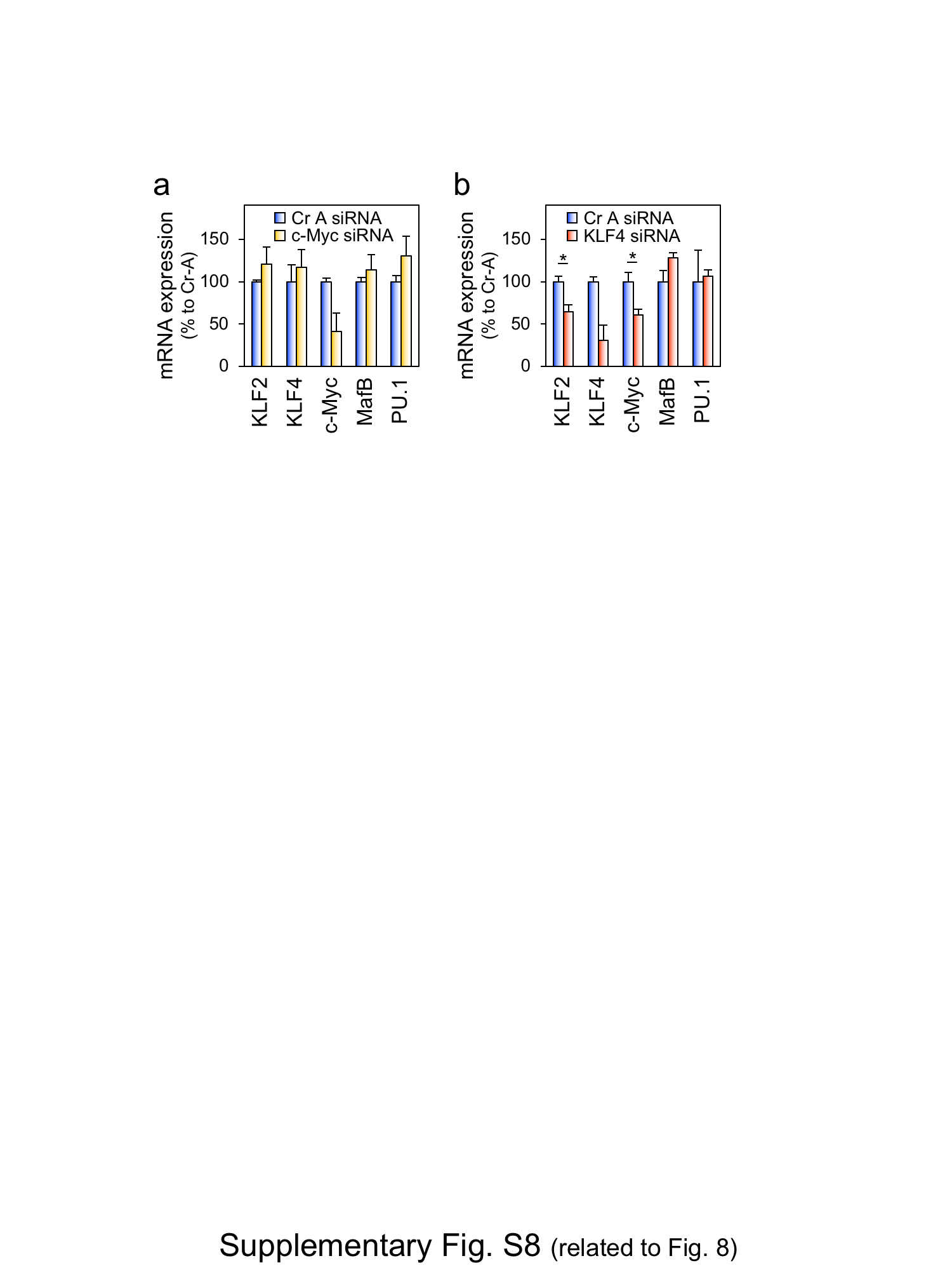

Supplement: Supplementary file 9 — Supplemental Figure S8 [file 41420_2020_300_MOESM9_ESM.png]
